# Evaluation of efficacy and safety of *Lacticaseibacillus rhamnosus* LRa05 in the eradication of *Helicobacter pylori*: a randomized, double-blind, placebo-controlled trial

**DOI:** 10.3389/fimmu.2024.1450414

**Published:** 2024-08-21

**Authors:** Yue Niu, Jing Li, Hongwei Qian, Chunli Liang, Xinyi Shi, Shurui Bu

**Affiliations:** ^1^ Department of Gastroenterology, Jinshan Hospital, Fudan University, Shanghai, China; ^2^ Department of General Practice, Shihua Community Health Service Center in Jinshan District, Shanghai, China

**Keywords:** *Helicobacter pylori*, eradication, bismuth quadruple therapy, *Lacticaseibacillus rhamnosus* LRa05, gut microbiota

## Abstract

**Aim:**

This study aims to evaluate the efficacy of *Lacticaseibacillus rhamnosus* LRa05 supplementation in enhancing *Helicobacter pylori* (*H. pylori*) eradication rate and alleviating the gastrointestinal side effects associated with bismuth quadruple therapy.

**Methods:**

*H. pylori*-positive patients were randomized to receive levofloxacin-based bismuth quadruple therapy combined either probiotic LRa05 or a placebo for two weeks, followed by LRa05 (1 × 10^10^ CFU) or maltodextrin for the next two weeks. *H. pylori* infection was detected by ^13^C breath test pre- and post-treatment. Blood and stool samples were collected at week 0 and week 4 for routine and biochemical analysis, and serum inflammatory markers. Gastrointestinal symptoms were evaluated using the gastrointestinal symptom rating scale (GSRS). Intestinal microbiota was analyzed using 16S rRNA sequencing. The research was listed under the Chinese Clinical Trial Registry (ChiCTR2300072220), and written informed consent was obtained from all participants.

**Results:**

The LRa05 group exhibited a trend toward higher *H. pylori* eradication rates (86.11%) compared to the placebo group (82.86%), though the difference was not statistically significant. Significant reductions in neutrophil count, alanine aminotransferase, aspartate aminotransferase, pepsinogen I, interleukin-6 (IL-6), tumor necrosis factor α (TNF-α) (*p* < 0.05) suggest that LRa05 supplementation may mitigate inflammation, enhance liver function, and potential aid in early cancer prevention. GSRS symptom scores showed that LRa05 alleviated abdominal pain, acid reflux, bloating, and diarrhea, enhancing patient compliance. Furthermore, 16S rRNA sequencing showed that LRa05 countered the antibiotic-induced disruption of gut microbiota diversity, primarily by increasing beneficial bacteria.

**Conclusion:**

Although LRa05 did not significantly improve the success rate of *H. pylori* eradication therapy, it has the potential to improve liver function and reduced levels of inflammatory markers such as IL-6 and TNF-α in the body, regulating the inflammatory response. In addition, it played a positive role in alleviating the adverse symptoms and gut microbiota disturbances caused by eradication therapy, providing a possible way to improve the overall health of patients and demonstrating promising clinical potential.

**Clinical Trial Registration:**

http://www.chictr.org.cn, identifier ChiCTR2300072220.

## Introduction

*Helicobacter pylori* (*H. pylori*) is widely recognized as a human pathogen (1). It has been linked to numerous gastrointestinal disorders including chronic active gastritis, peptic ulcers, mucosa-associated lymphoid tissue (MALT) lymphoma (2) and stomach cancer. Furthermore, research has indicated that *H. pylori* is not only associated with gastric diseases, but also with a series of extra-gastric conditions (3), such as vitamin B12 deficiency, iron deficiency anemia, non-alcoholic fatty liver disease, idiopathic thrombocytopenic purpura, coronary atherosclerosis, Alzheimer’s disease, etc. A meta-analysis report indicates that the estimated global prevalence of *H. pylori* infection has decreased from 58. 2% (95% CI 50.7 - 65.8) during the period from 1980 to 1990 to 43.1% (40.3 - 45.9) during the period of 2011-2022 (4). Although the prevalence of *H. pylori* infection has decreased in the past decade, it still remains at a relatively high level. Consequently, the prevention and treatment of *H. pylori* -related diseases continue a prominent topic in the field of gastroenterology, controlling *H. pylori* poses a significant public health and global societal challenge.

It is well known that intestinal microbiota stability is crucial for human health. However, several studies have demonstrated that eradicating *H. pylori* may alter the gastric and intestinal microbial milieu in the short term (5, 6). In patients who have been unsuccessful in eliminating *H. pylori*, there will inevitably be alterations in the intestinal microbiota following one or even multiple eradication treatments (7). The inclusion of probiotics on the basis of bismuth quadruple therapy represents a novel and effective therapy (8, 9). Investigations have demonstrated that probiotic-assisted therapy can construct a beneficial profile of gut microbiota (10, 11). Nevertheless, there exists a variety of probiotics, and there is no uniform consensus on the timing for combining them with *H. pylori* eradication drugs (whether before, after, or simultaneously). Additionally, while it is generally agreed upon that probiotics can mitigate gastrointestinal adverse effects associated with eradication therapy (12, 13), further investigation is warranted to validate this claim.

The bismuth quadruple regimen is currently the recommended primary empirical treatment plan against *H. pylori* infections by consensus (14, 15). While it is effective for most infections produced by this bacterium, there are a number of challenges that lead to an unsatisfactory eradication rate (16), including the emergence of antibiotic-resistant strains (17), increased adverse reactions, and decreased host CYP2C19 gene polymorphism. Furthermore, widespread and irregular use of antibiotics may result in various effects, such as antibiotic-associated diarrhea (AAD), toxic side effects, and the short-term selection and spread of drug-resistant bacteria. In the long term, it may also result in alterations to the mucosal immune response pattern, the induction of gastric and intestinal microbiota imbalance (18), and the potential contribution to the development of certain diseases. In conclusion, these issues present significant difficulties in clinical *H. pylori* treatment and highlight the urgent need for improved therapeutic strategies.

Some probiotics such as *Bifidobacterium, Lactobacillus crispatus* FSCDJY67L3 and *Lactobacillus reuteri* DSM 17648 have been used in clinical studies related to the eradication of *H. pylori* (13, 19–23). In these relevant clinical trials, there is controversy about whether probiotics supplementation can improve the eradication rate of *H. pylori*. Some studies believe that probiotics supplementation can improve the eradication rate, while others hold the opposite attitude. However, most articles believe that probiotics supplementation can effectively alleviate the adverse gastrointestinal symptoms caused by eradication therapy, which is also the view supported by our study.

*Lacticaseibacillus rhamnosus* LRa05, originating from the feces of healthy infants, has been extensively utilized in preclinical studies, *in vitro* tests, and animal models (24–28). Research indicates that LRa05 can effectively regulate the gut microbiota, improving glucose metabolism (29) and alleviating the lipid accumulation of high-fat diet mice (27), and its safety has been confirmed by studies. It is considered to be a potential probiotic for the food industry (28), but few articles have used LRa05 for *H. pylori* eradication therapy. Therefore, in this study, we employed LRa05 as a supplementary treatment for *H. pylori* eradication, comprehensively evaluating its impact on eradication rates, blood biochemistry parameters, inflammatory markers, adverse gastrointestinal symptoms (such as nausea, vomiting, diarrhea, dyspepsia, and other symptoms), and changes in the gut microbiome.

## Materials and methods

### Study design and ethical statement

**Figure 1:**
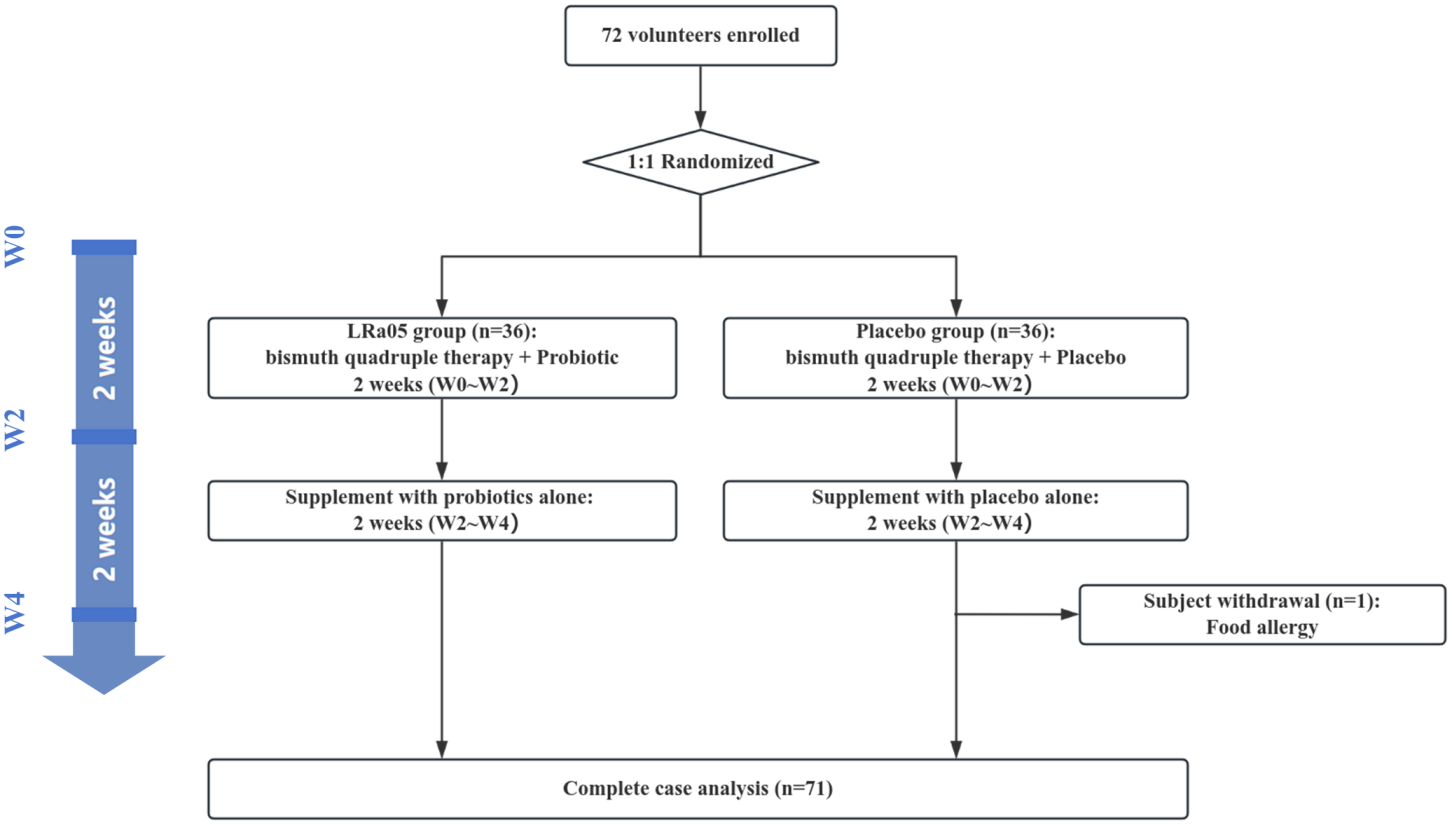
Flow diagram showing the study design. LRa05, *Lacticaseibacillus rhamnosus*.

**Figure 2:**
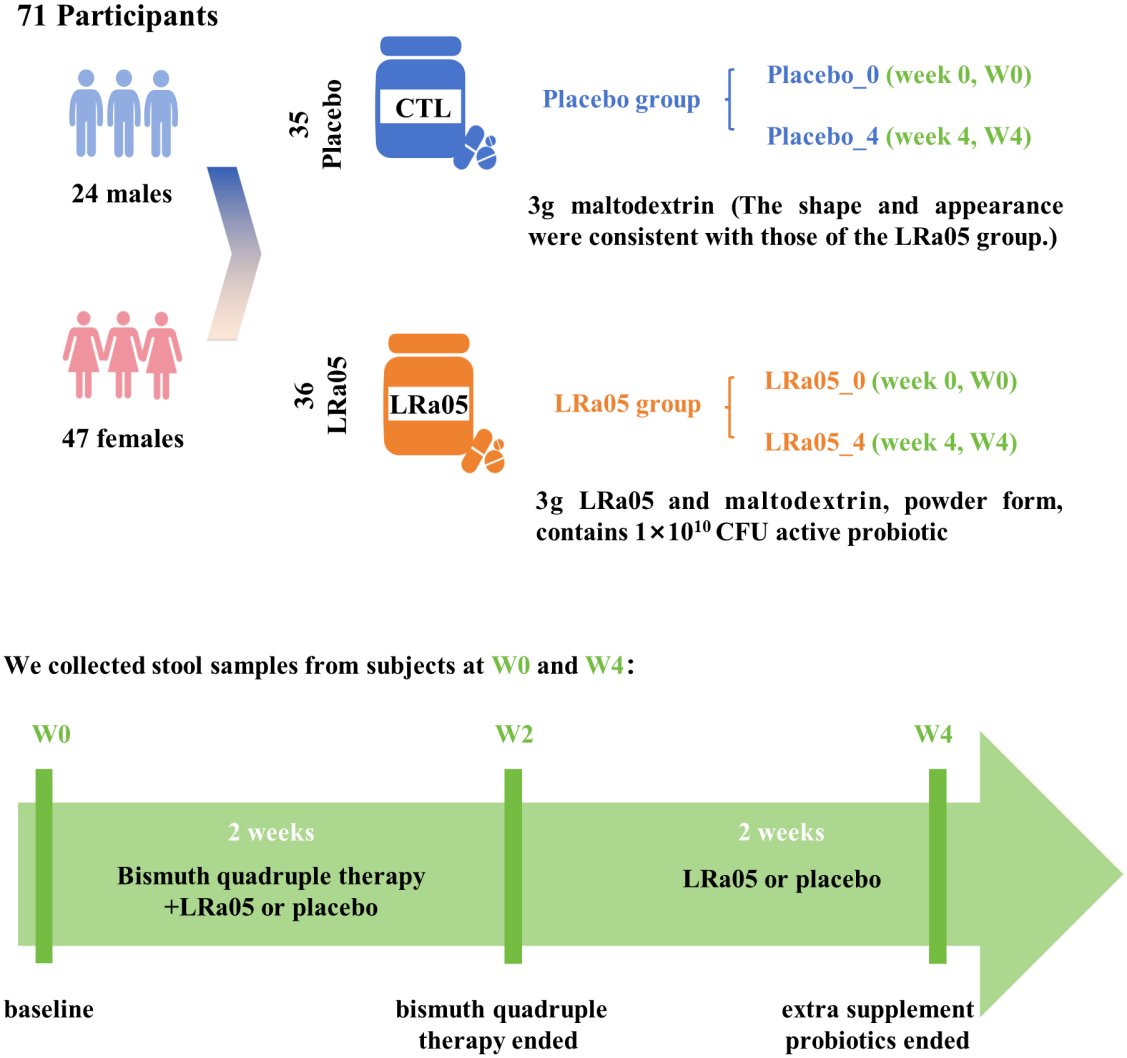
Study design details. Seventy-two subjects were recruited and 71 participants completed the trial. We collected stool and blood samples at baseline (W0) and 4 weeks after the intervention (W4), and conducted questionnaires. LRa05, *Lacticaseibacillus rhamnosus*.

This study utilized a double-blind, placebo-controlled design conformant with the Declaration of Helsinki principles. We enrolled 72 patients infected with *H. pylori* from the outpatient department of Jinshan Hospital. Subjects were randomly assigned in a 1:1 ratio to either the LRa05 group or the placebo group. The overview of the trial design and process is illustrated in **Figures 1** , **2**. This trial was conducted at Jinshan Hospital affiliated to Fudan University in China from June 2023 to April 2024.

### Population recruitment

Only individuals who fulfill the following criteria were eligible for selection: (1) Written informed consent has been obtained, ensuring that patients have a comprehensive understanding of the experiment’s content, procedures, and potential adverse reactions; (2) Subjects (including males) must not have any family planning intentions and should voluntarily adopt effective contraceptive measures from 14 days prior to screening until 6 months after completion of the trial; (3) Patients aged between 18 and 65 years old are included; (4) Male volunteers must weigh not less than 50 kg, while female subjects should have a minimum weight of 45 kg with a BMI of ≥ 19.0 kg/m²; (5) *H. pylori* infections were diagnosed using either ^13^C/^14^C-UBT or gastric mucosal tissue immunohistochemical detection of Hp (IHC for Hp). The exclusion criteria were determined as follows: (1) Contraindication or allergy to any component of the study drugs; (2) Substance abuse (drug or alcohol, consuming more than 14 units of alcohol per week: 1 unit = 285 mL of beer, 25 mL of spirits, or 100 mL of wine); (3) A history of prior *H. pylori* eradication therapy; (4) Use of antibiotics or probiotics within the preceding 3 months; (5) Presence of severe concurrent organic disease such as gastrointestinal bleeding, obstruction, perforation or tumor; (6) Patients with liver disease indicated by an aminotransferase index exceeding 1.5 times the upper limit of normal and kidney disease indicated by a creatinine index surpassing the upper limit of normal; (7) Positive fecal occult blood test results; (8) Participants with a severe mental illness impeding collaboration and communication necessary to meet study requirements; (9) Pregnant or breastfeeding women; (10) Individuals deemed unsuitable by investigators.

### Intervention and monitoring protocol

Participants were administered a standard antibiotic regimen comprising Rabeprazole (20 mg bid), Levofloxacin (250 mg bid), Metronidazole (400 mg bid), and Bismuth pectin (200 mg tid). Additionally, individuals in the LRa05 group received *Lacticaseibacillus rhamnosus* LRa05 (1 × 10^10^ CFU), whereas those in the placebo group were given a placebo consisting of maltodextrin, 1 packet of 3 g per day (**Figure 2**). Both the probiotic and placebo products were supplied by Wecare Probiotics Co., Ltd, ensuring consistency and quality control throughout the study. The intervention spanned four weeks, with the first two weeks involving combined treatment of antibiotics and probiotic/placebo, followed by a two-week period where only probiotic LRa05 or placebo were administered.

### Outcomes

The primary outcome is the eradication rate. The secondary outcome measures of this study encompassed the impact of combined probiotic LRa05 on blood routine examination, serum biochemical index, the total Gastrointestinal Symptom Rating Scale (GSRS) score, the incidence of adverse reactions, to evaluate compliance and security levels. Additionally, we assessed alterations in intestinal microbiota, including short-term variations in microbiota diversity (at week 0 and week 4), modifications in gut microbiota.

### *H. pylori* and hematology detection

We assisted patients with confirmed *H. pylori* infection and inclusion in the study by either of the following methods: gastric antrum mucosal tissue immunohistochemical detection of Hp (IHC for Hp) positive, ^13^C-UBT positive, or ^14^C-UBT positive. IHC for Hp were performed after gastric mucosal tissue was obtained by gastroscopy (30). And the ^13^C/^14^C-UBT test is simpler and more convenient, requiring the patient to take orally an isotopically labeled urea solution and blow at regular intervals (31). In addition, we collected blood samples from patients, sent the samples to the laboratory for blood routine (such as RBC: red blood cell, NEUT: neutrophil, PLT: platelet, HGB: hemoglobin) and blood biochemical tests (including AST: spartate aminotransferase, ALT: alanine aminotransferase, TBIL: total bilirubin, UREA: urea, CREA: creatinine) (Mindray BS-2800M, China). We also detected the level of blood inflammatory factors (IL-6: Interleukin-6, TNF-α: Tumor Necrosis Factor-alpha, IL-17: Interleukin-17, IL-8: Interleukin-8), gastrin-17 (GT17), and pepsinogen I (PGI) by ELISA method. All measurements were performed according to the manufacturer’s instructions (Jiangsu Meimian industrial Co., Ltd Yancheng).

### Fecal sample collection

Fresh stool samples were collected from all patients at baseline week 0 (W0) and at week 4 (W4). Participants were instructed to promptly deliver the fecal specimen to the research assistant within the hospital on the day of collection. Subsequently, all stool samples were immediately frozen and stored at a temperature of -80°C for preservation.

### The 16s RNA sequencing

The total genomic DNA of the fecal samples was isolated using the QIAamp Fast DNA Stool Mini Kit (Qiagen, Hilden, Germany) according to the manufacturer’s instructions. The extracted DNA was kept in a refrigerator at a temperature of -80°C until processed. Polymerase chain reaction (PCR) was used to amplify the V3V4 16s rRNA bacterial gene. Upstream (5’-CCTACGGGNGGCWGCAG-3’) and downstream (5’-GACTACHVGGGTATCTAATCC-3’) primer, bacterial genomic template DNA and Premix Taq constitutes the PCR reaction system. The amplifications were carried out using the program: initial denaturation at 95°C for 3min, followed by 25 cycles of denaturation at 94°C for 15s, annealing at 55°C for 30s, and extension at 72°C for 45s, with a final elongation step at 72°C for 10min. The PCR products were combined in equal proportions and subsequently purified using the Qiagen Gel Extraction Kit (Qiagen, Germany) to ensure uniformity and quality for downstream applications. The amplified DNA fragments were subsequently subjected to sequencing using an Illumina MiSeq platform (Illumina, San Diego, USA). After the sequencing was completed, the software cutadapt v2.6 was used to filter the raw date according to the window to remove the low quality, and the clean date was obtained. The filtered high-quality clean data was used for subsequent analysis. Reads were spliced into tags according to the overlap between reads, and tags were grouped into OTU, compared with the database and annotated by species. Based on the OTU and annotated results, sample species complexity analysis, inter-group species difference analysis and association analysis were carried out.

### Microbiota analysis

The Bray-Curtis distance analysis from the vegan package in R (4.2) was utilized to evaluate the ecological diversity between the placebo and LRa05 groups based on the relative abundance of bacterial genera. Chao1 and ACE, Shannon and Simpson indices were used to evaluate richness and diversity of gut microbiota in participants following the intervention with LRa05. β diversity was assessed using Bray-Curtis distance, and the results were visualized through principal coordinates analysis (PCoA) plot.

### Statistical analysis

Statistical analyses were conducted using SPSS software, version 27.0. For this study, quantitative data that conformed to a normal distribution are expressed as mean ± standard deviation (SD). Results were analyzed using one-way analysis of variance (ANOVA), followed by Tukey’s multiple comparison test to determine statistical significance. Quantitative data not following a normal distribution are presented as median and interquartile range. Nonparametric tests were used for analysis, with between-group comparisons performed using the Mann-Whitney U test, and within-group comparisons using the Wilcoxon signed-rank test. Categorical data were compared using the chi-square test. Correlation between two variables was assessed using Fisher’s exact test. The level of significance was set at α = 0.05, with *p* < 0.05 indicating statistically significant differences.

## Results

### Baseline characteristics of the subjects

Of the 72 participants enrolled, 71 completed the trial. Unfortunately, one participant in the placebo group was withdrawn early due to sea-food allergy (**Figure 1**). Ultimately, we collected stool and blood samples from 71 patients (**Figure 2**). The baseline information of enrolled patients is shown in **Table 1**. There were no significant differences in gender composition, age, BMI, occupation, marital status, smoking habits and medical history between the two groups (*p* > 0.05). Therefore, we believe that the biochemical indicators, microbiota composition, the GSRS score and other test indicators of the two groups of patients are comparable.

**Table 1:** Baseline characteristics of participants in the two groups.

Characteristics	Placebo (n=35)	LRa05(n=36)	*p* value
Gender			0.932
Male	12 (34.29%)	12 (33.33%)	
Female	23 (65.71%)	24 (66.67%)	
Age ^a^	45.23 ± 11.95	48.03 ± 9.61	0.486
BMI ^b^	22. 86 (21.78~26.33)	22.27 (20.96~24.24)	0.173
Job			0.307
Manual worker	1 (2.86%)	0	
Mental laborers	34 (97.14%)	36 (100%)	
Marriage			0.307
Unmarried	1 (2.86%)	0	
Married	34 (97.14%)	36 (100%)	
Smoking	0 (100%)	0 (100%)	≈1
Medical History	0 (100%)	0 (100%)	≈1

a Data are presented as mean ± standard deviation (SD).

b Data are presented as median (P_25_, P_75_).

LRa05, *Lacticaseibacillus rhamnosus*.

### *H. pylori* eradication rate and adverse events

**Figure 3:**
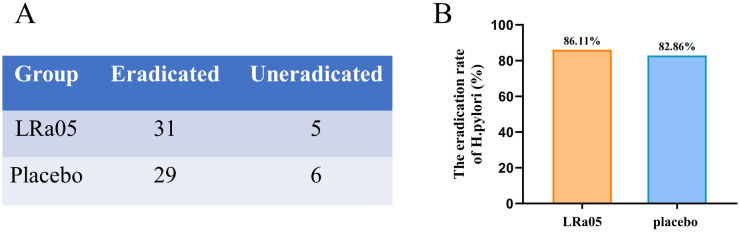
Eradication rate of *H. pylori* in patients treated with probiotics or placebo. The number of people in both groups with or without *H. pylori* eradication after treatment **(A)**. The LRa05 group had a higher eradication rate than the placebo group **(B)**, but there was no significant difference in the eradication rate between the two groups (Chi square = 0.144, *p* = 0.7048). LRa05, *Lacticaseibacillus rhamnosus*.

The *H. pylori* eradication rates of the LRa05 and placebo group were 86.11% and 82.86% (**Figure 3**), respectively. This indicates that probiotics could improve the eradication rate to a certain extent and that probiotics would not have a negative impact on the eradication therapy. However, there was no statistically significant difference (χ2 = 0.144, *p* = 0.7048).

### Changes in biochemical indicators before and after intervention

**Figure 4:**
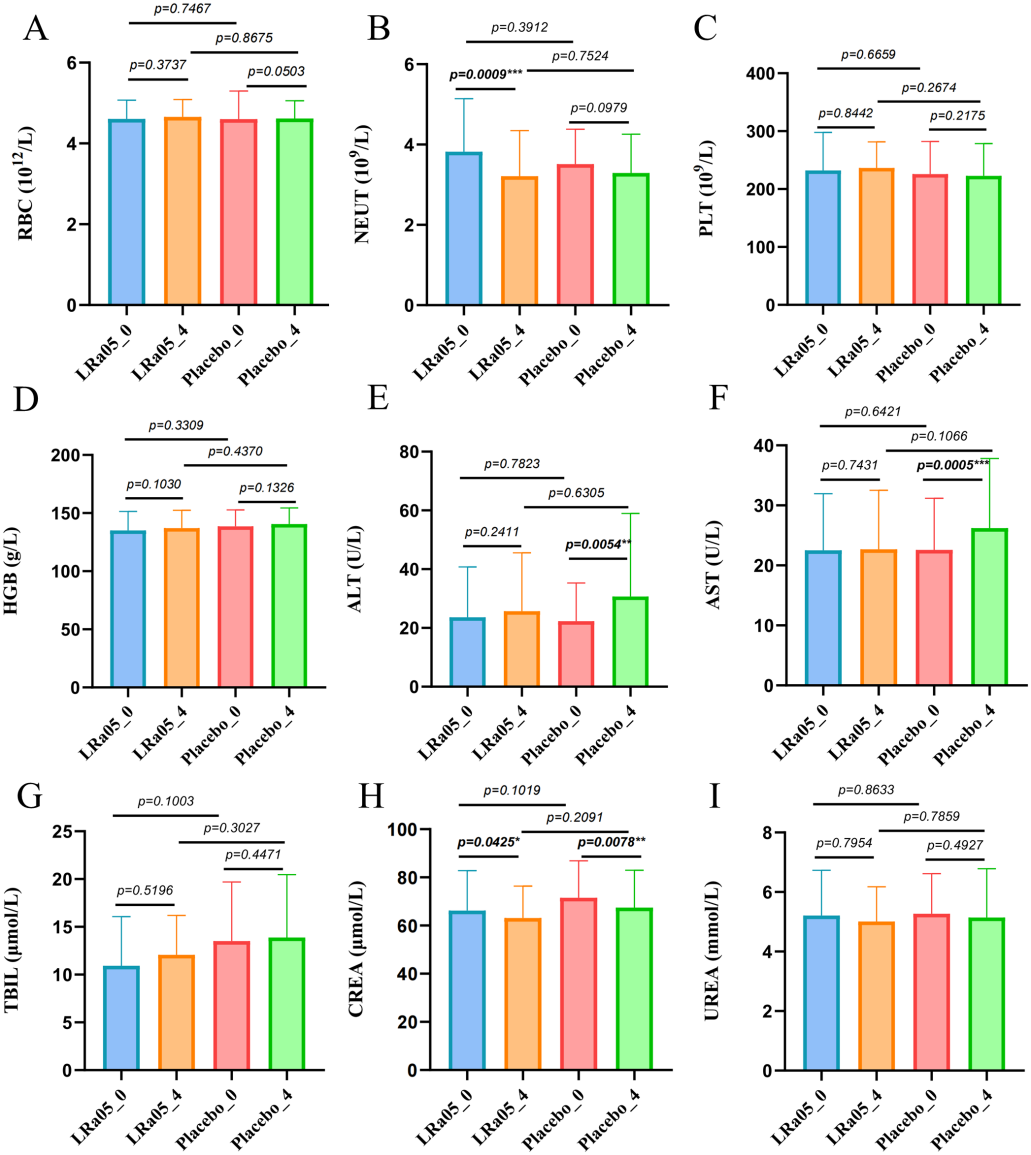
Results of statistical analysis of blood biochemical indexes of patients. **(A)** RBC, red blood cell, **(B)** NEUT, neutrophil, **(C)** PLT, platelet, **(D)** HGB, hemoglobin, **(E)** ALT, alanine aminotransferase, **(F)** AST, spartate aminotransferase, **(G)** TBIL, total bilirubin, **(H)** CREA, creatinine, **(I)** UREA, urea. LRa05, *Lacticaseibacillus rhamnosus*. _0: week 0, _4: week 4. **p<0.01, ***p<0.001.

**Figure 5:**
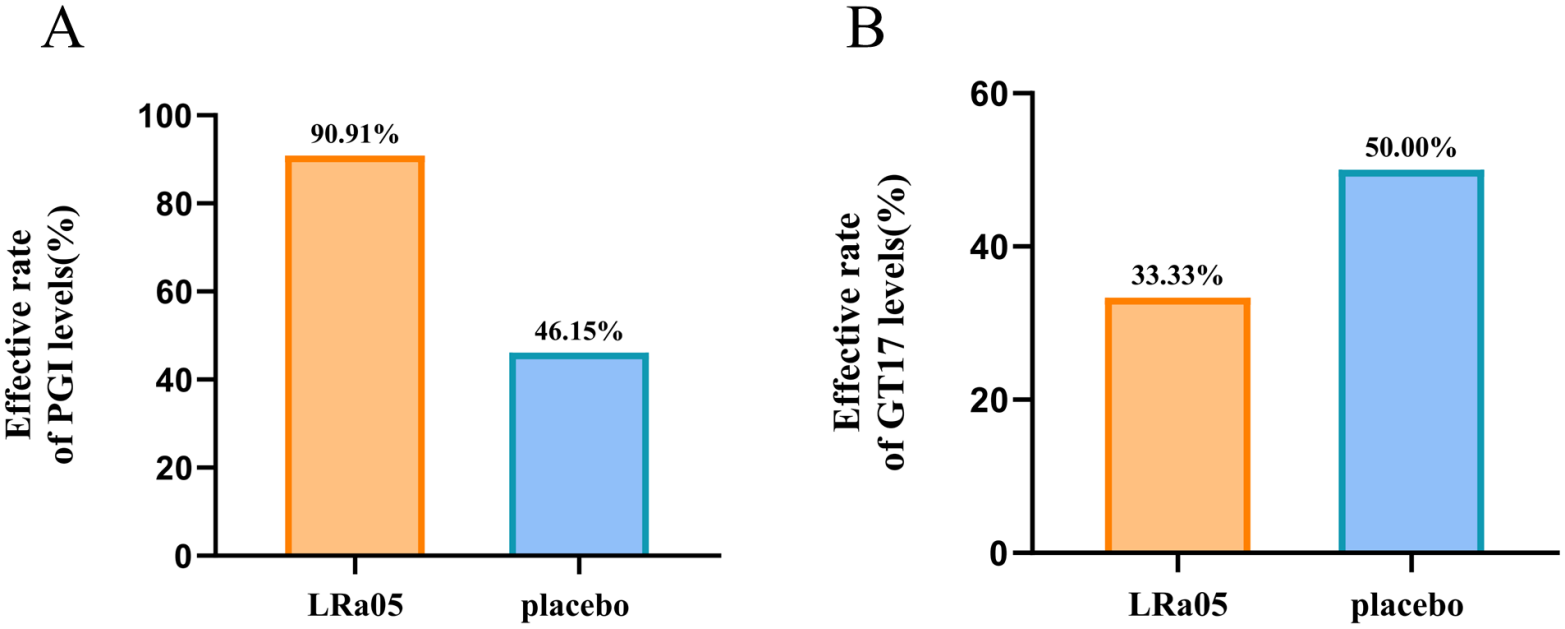
Changes of PGI levels in patients. The formula for calculating the effect rate of PGI level: (week0 number of abnormal PGI indicators - week4 number of abnormal PGI indicators)/week0 number of abnormal PGI indicators. GT17, gastrin-17; PGI, pepsinogen I; LRa05, *Lacticaseibacillus rhamnosus*.

NEUT showed a significant reduction in the LRa05 group before and after treatment (*p* = 0.0009), while there was no significant difference in the placebo group. Similarly, ALT and AST in the placebo group changed significantly before and after treatment (*p* = 0.0054, *p* = 0.0005, respectively), while the difference was not significant in the LRa05 group (**Figure 4**). Compared with the placebo group, the LRa05 group is more effective in regulating the level of PGI (*p* = 0.033, **Figure 5**).

### Regulation of serum inflammatory factors

**Figure 6:**
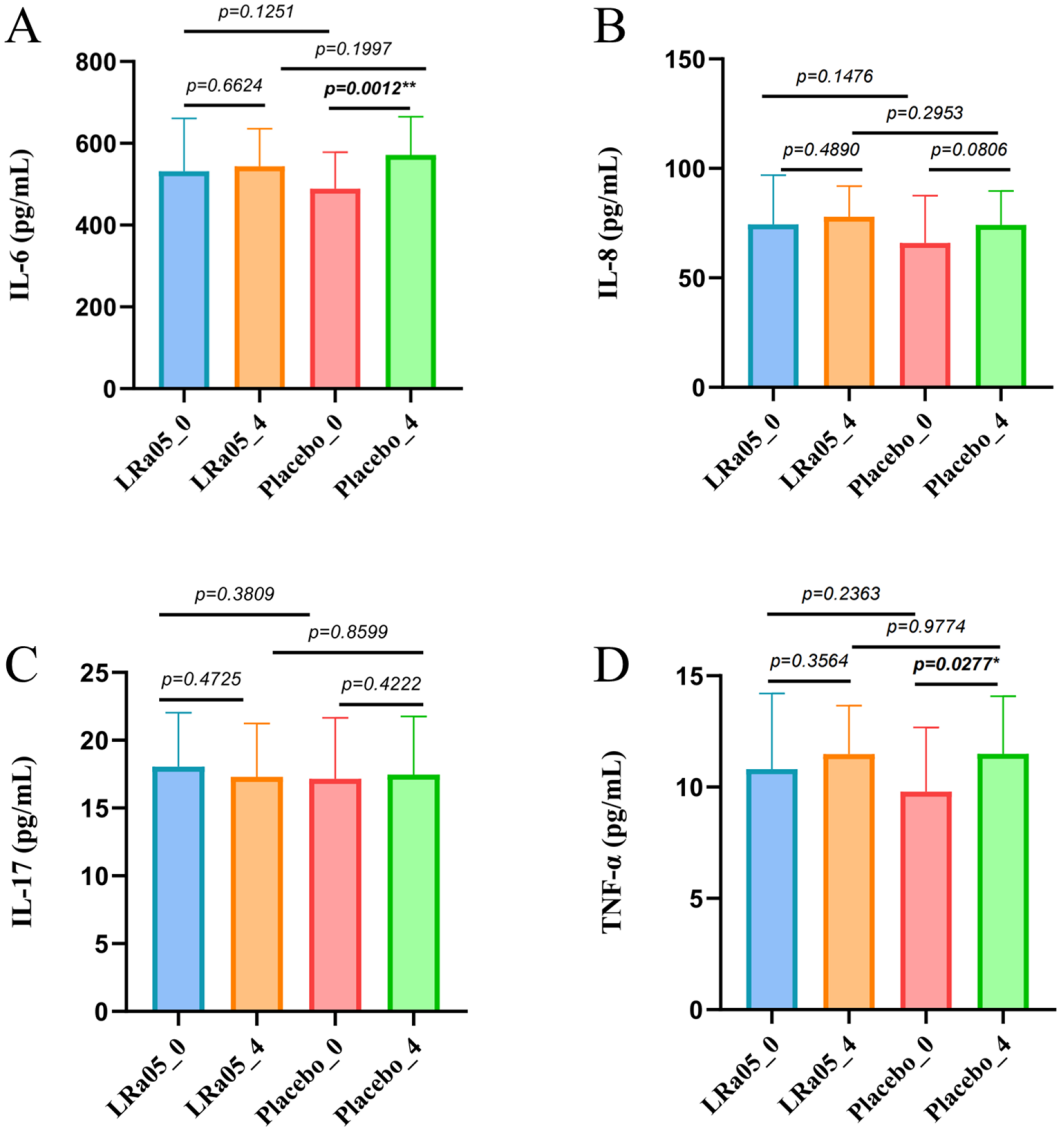
Serum inflammatory marker dynamics in patients. **(A)** Interleukin-6 (IL-6), **(B)** Tumor Necrosis Factor-α (TNF-α), **(C)** Interleukin-17 (IL-17), **(D)** Interleukin-8 (IL-8). LRa05, *Lacticaseibacillus rhamnosus*. Changes measured at Week 0 (_0) and Week 4 (_4) for both LRa05 and placebo groups. *p<0.05, **p<0.01.

The comparison of inflammatory markers showed that the levels of IL-6 (*p* = 0.0012) and TNF-α (*p* = 0.0277) in the placebo group significantly increased after treatment, and the level of IL-8 also increased significantly, though not to a statistically significant level (*p* = 0.081) (**Figure 6**). However, there were no significant changes in the levels of these inflammatory factors in LRa05 group (*p* > 0.05).

### Adverse gastrointestinal symptoms

The gastrointestinal adverse symptoms in the patients were assessed using the GSRS. Measurements were taken at baseline (W0) and four weeks post-intervention (W4). The analysis demonstrated a significant reduction in GSRS scores from baseline to follow-up in both the placebo and LRa05 group (**Table 2**). A more granular analysis indicated that, relative to the placebo, the LRa05 group was significantly more effective in alleviating gastrointestinal symptoms, including abdominal pain, acid reflux, abdominal distension, and loose stools (*p* < 0. 05, **Table 3**).

**Table 2:** Efficacy of patients with improved GSRS scores.

GSRS Score	LRa05 group (n = 36)	Placebo group (n = 35)	*p* value
week 0	6.61 ± 5.42	7.00 ± 3.99	0.4257
week 4	3.69 ± 3.61	4.89 ± 5.33	0.3728
*p* value	0.0006***	0.0058**	–

**p < 0.01, ***p < 0.001. LRa05: *Lacticaseibacillus rhamnosus*.

**Table 3:** Subcategorize gastrointestinal symptoms.

gastrointestinal symptoms	LRa05 group	Placebo group	*p* value
abdominal pain	91.67%(11/12)	36.36%(4/11)	0.009^**^
acid reflux	73.33%(11/15)	33.33%(5/15)	0.028^*^
abdominal distension	60.00%(12/20)	28.57%(6/21)	0.043^*^
loose stools	50.00%(9/18)	13.33%(2/15)	0.026^*^

*p < 0.05, **p < 0.01. LRa05, *Lacticaseibacillus rhamnosus*. The proportion in the table refers to the proportion of people with gastrointestinal symptoms in remission.

### Effects of the intervention on gut microbiota diversity

**Figure 7:**
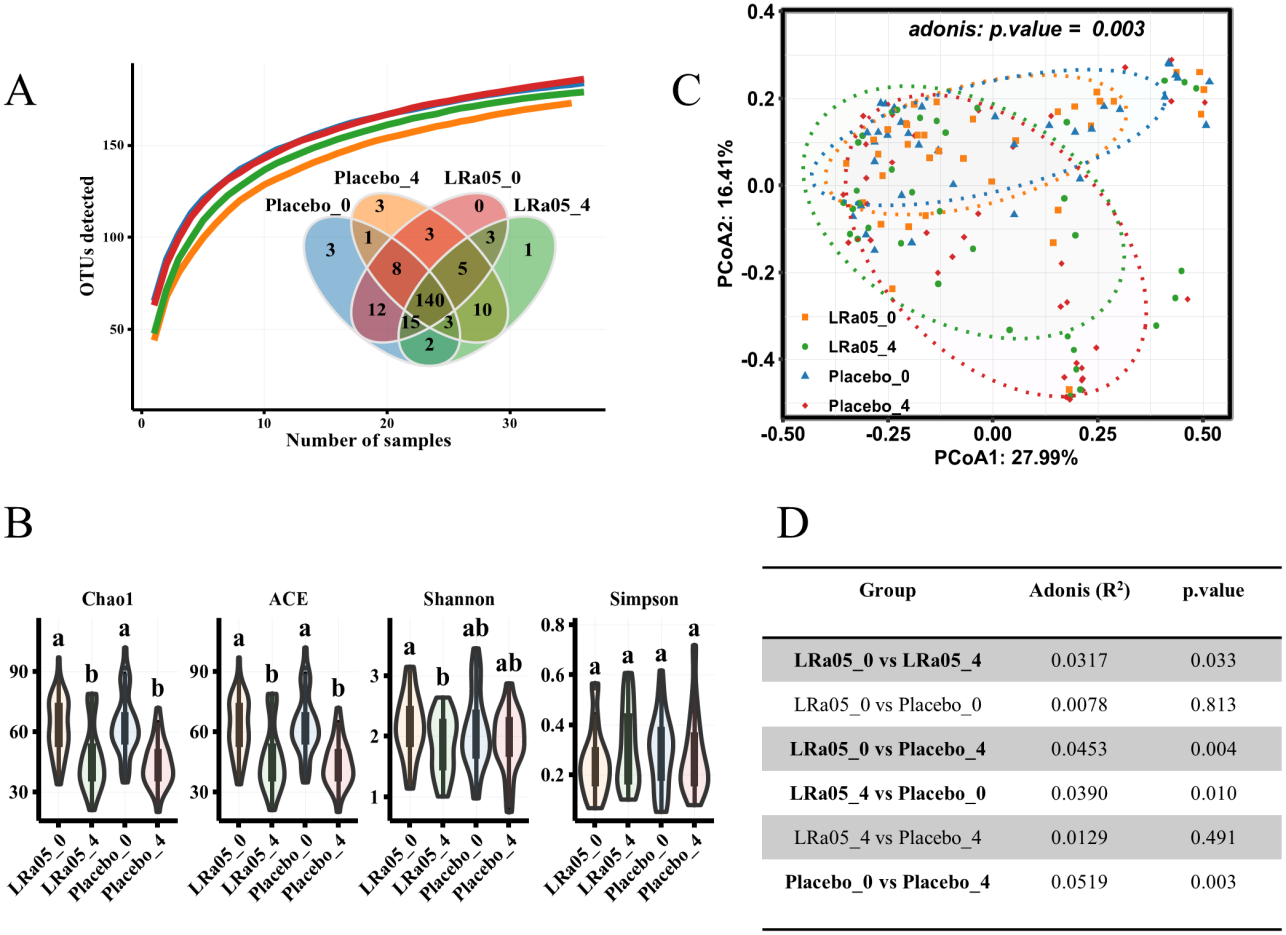
Changes in gut microbiota diversity pre- and post-intervention. **(A)** Species accumulation curve and Venn diagram depicting species distribution across placebo and LRa05 groups. **(B)** α diversity alterations measured pre- and post-intervention. **(C)** β diversity changes evaluated using principal component analysis based on the Bray–Curtis distance, with each point representing an individual subject’s gut microbiota composition. **(D)** Statistical comparison of β diversity differences between groups. LRa05, *Lacticaseibacillus rhamnosus.* _0: week 0, _4: week 4. All the averages are listed in order from largest to smallest, and then the largest average is marked with the letter a; This average is compared with the following averages, where the difference is not significant, marked with the letter a, until a certain average with a significant difference, marked with the letter b. Where there is an identical marking letter, the difference is not significant, and where there is a different marking letter, the difference is significant.

The species accumulation curve (**Figure 7A**) shows a continuous increase in the number of species in each group, indicating that species accumulation has reached saturation. In addition, the Venn diagram in **Figure 7A** shows 140 common species between the placebo group and the LRa05 group, accounting for 67% of the 209 species found. Further analysis (**Figure 7B**) showed that both placebo and LRa05 intervention reduced the species richness of intestinal microbiota (Chao1 and ACE), and did not cause significant alterations in community diversity (Shannon and Simpson). Furthermore, for the Shannon index, the LRa05 intervention significantly decreased, while there was a decrease in the placebo group, but the change was small and not statistically significant. These findings are consistent with our species accumulation analysis and the results of the Venn Diagram.

Although α diversity analysis did not reveal significant changes in gut microbiota resulting from placebo and LRa05 interventions, β diversity analysis showed (**Figure 7C**) that placebo and LRa05 interventions had significant effects on gut microbiota composition (*p* = 0. 003). The adonis assay was used to assess the effects of LRa05 and placebo interventions on gut microbiota composition. However, at the end of the study, the difference between the LRa05 and placebo groups was not significant (*p* = 0. 491) (**Figure 7D**).

### Changes at the phylum and genus levels in intestinal microbiota

**Figure 8:**
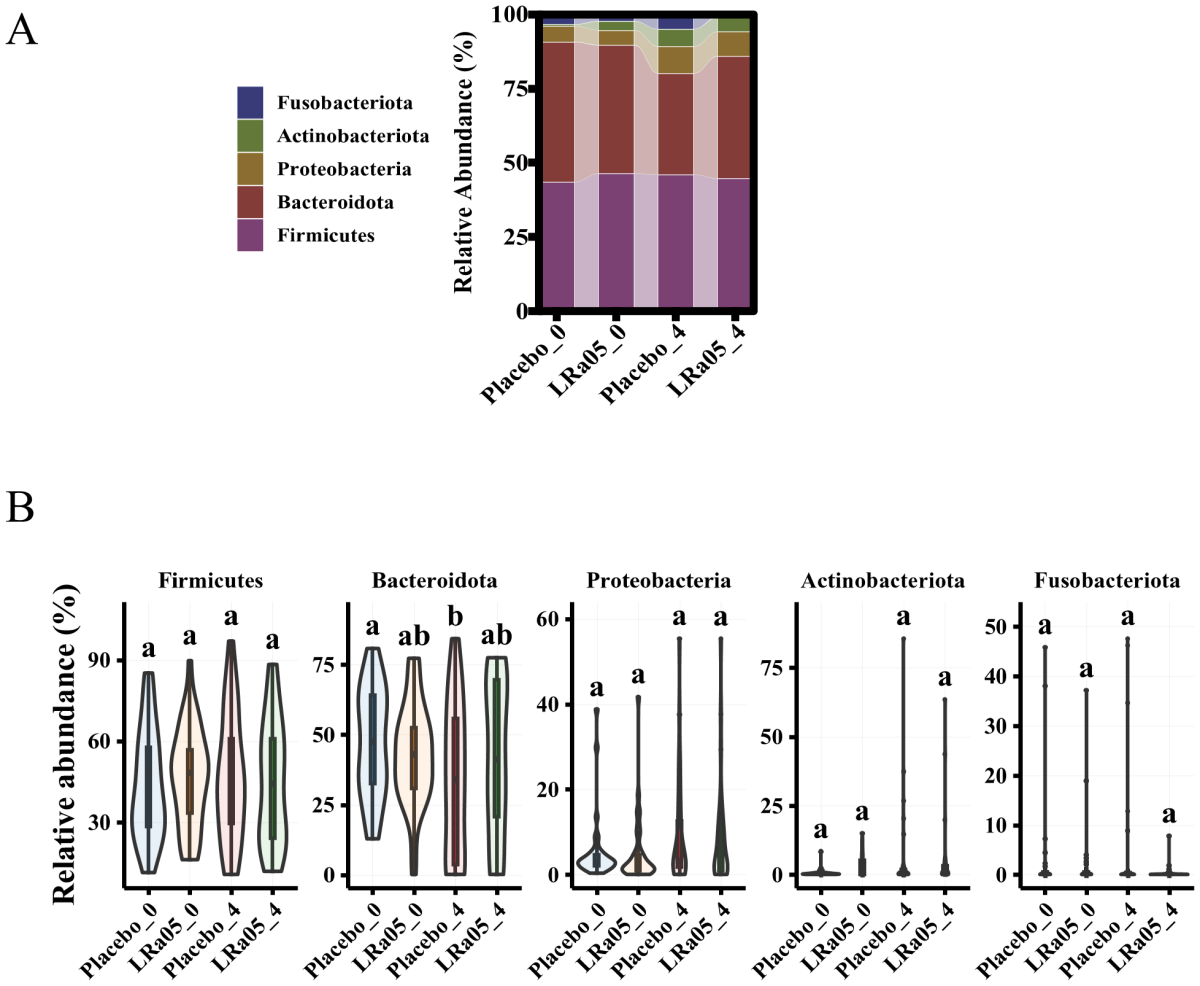
Changes in gut microbiota composition in placebo and LRa05 groups pre- and post-intervention. **(A)** Distribution of microbiota abundance at the phylum level across placebo and LRa05 groups. **(B)** Changes in phylum-level abundance from baseline (W0) to post-intervention (W4). LRa05, *Lacticaseibacillus rhamnosus*, _0: week 0, _4: week 4. All the averages are listed in order from largest to smallest, and then the largest average is marked with the letter a; This average is compared with the following averages, where the difference is not significant, marked with the letter a, until a certain average with a significant difference, marked with the letter b. Where there is an identical marking letter, the difference is not significant, and where there is a different marking letter, the difference is significant.

At the phylum level, Firmicutes, Bacteroidetes, Proteobacteria, Fusobacteriota, and Actinobacteria are the five most dominant bacterial phyla (accounting 99.9%) in the intestinal microbiota of the subjects (**Figure 8A**). Our analysis of the gut microbiota data at time points W0 and W4 revealed that there were no significant differences in the intestinal microbiota at the phylum level between the Placebo and LRa05 groups at any given time point (**Figure 8B**), except for Bacteroidetes. At the W4 point, the level of Bacteroidetes significantly decreased in the placebo group. Regarding Proteobacteria, both groups showed an upward trend at the end of the intervention period after antibiotic treatment.

In terms of genus classification, although most beneficial bacteria such as *Faecalibacterium*, *Roseburia* and *Bifidobacterium* have generally decreased in all patients after treatment, the reduction of these bacteria groups is less pronounced in patients receiving LRa05 intervention. For example, although the content of *Faecalibacterium* in the LRa05 group has decreased, the rate of decline in *Faecalibacterium* is significantly smaller than in the placebo group (*p* = 0.008). *Bacteroides* and *Blautia* genera in the LRa05 group also showed a decline, but to a similarly small extent compared to the placebo group, suggesting a protective effect of LRa05 intervention. The *Lachnospira* and *Roseburia* genera decreased significantly in the placebo group, whereas the decrease was more moderate in the LRa05 group. After antibiotic treatment, the abundance of both beneficial and harmful bacteria decreased, but the following bacteria groups increased in the LRa05 group compared to the placebo group: *Faecalibacterium, Lachnospira, Parabacteroides, Subdoligranulum, Phascolarctobacterium, Agathobacter, Fusicatenibacter, Alistipes, Coprococcus, Oscillibacter, Parasutterella, Megamonas* (**Supplementary Table 1**).

### Correlation analysis between gut microbiota and inflammatory markers after LRa05 intervention

**Figure 9:**
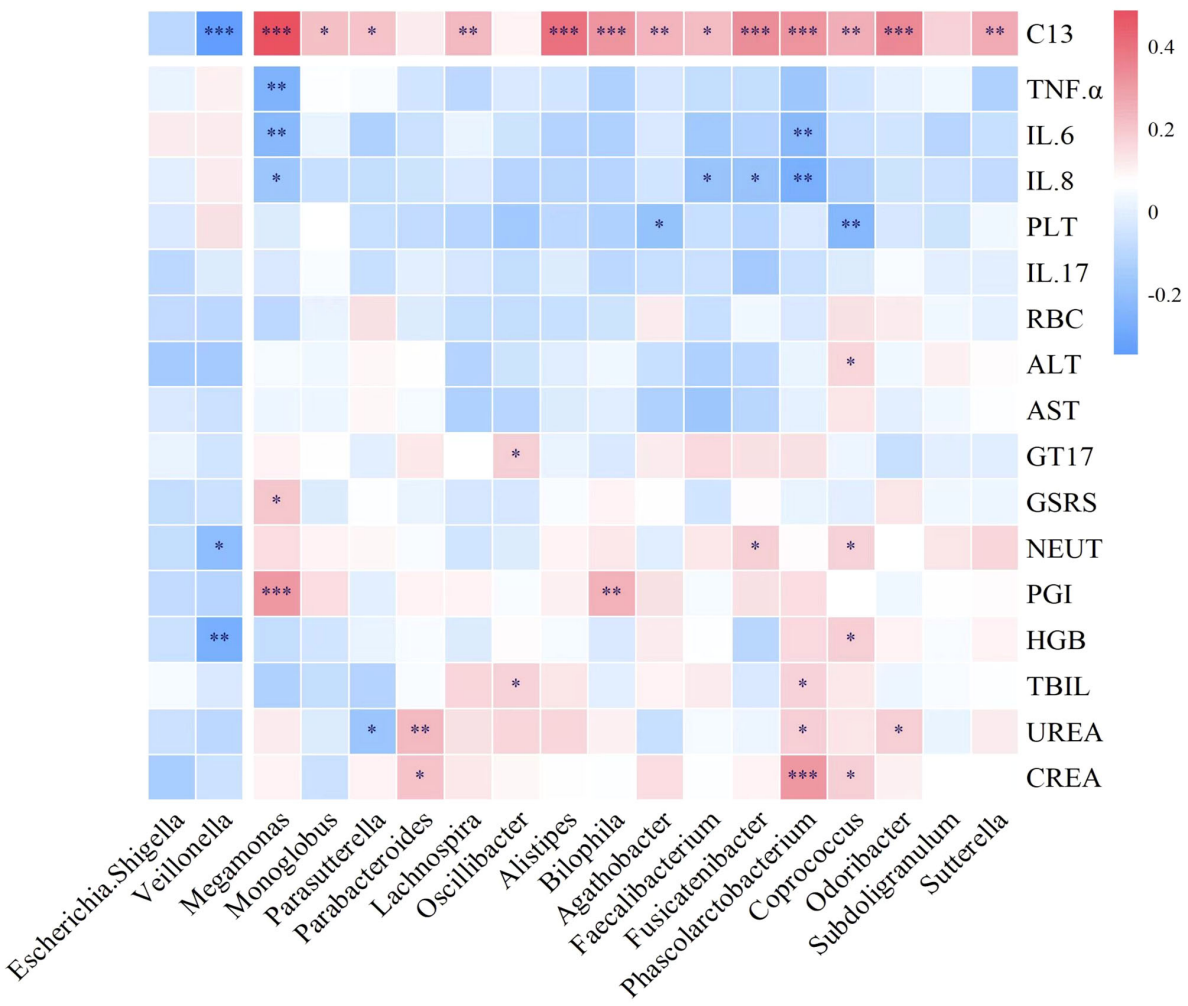
Correlation between intestinal microbiota and serum biochemistry, inflammatory factors and ^13^C. ^13^C, ^13^C-urea breath test; TNF-α, tumor necrosis factor α; IL-6, interleukin-6; IL-8, interleukin-8; PLT, platelet; IL-17, interleukin-17; RBC, red blood cell; ALT, alanine aminotransferase; AST, spartate aminotransferase; GT17, gastrin-17; GSRS, gastrointestinal symptom rating scale; NEUT, neutrophil; PGI, pepsinogen I; HGB, hemoglobin; TBIL, total bilirubin; UREA, urea; CREA, creatinine. *p<0.05, **p<0.01, ***p<0.001.

The *Megamonas* genus exhibits a positive correlation with PGI, GT17, and C13, while showing a negative correlation with inflammatory factors such as IL-6, IL-8, and TNF-α. *Phascolarctobacterium* has a positive correlation with indicators like CREA, UREA, TBIL, and C13, while displaying a negative correlation with inflammatory factors like IL-6 and IL-8. *Faecalibacterium* and *Fusicatenibacter* demonstrate a negative correlation with IL-8 and a positive correlation with C13. Therefore, LRa05 combination therapy adjusts the abundance of *Megamonas, Phascolarctobacterium, Faecalibacterium*, and *Fusicatenibacter* to reduce intestinal inflammation and promote intestinal health (**Figure 9**).

## Discussion

The results demonstrate that although LRa05 does not significantly enhance the *H. pylori* eradication rate, it effectively improves liver function, modulates inflammatory responses, and significantly optimizes gastrointestinal symptoms and dysbiosis caused by eradication treatment. This suggests that LRa05 can serve as a potent adjunct to conventional *H. pylori* eradication therapies, mitigating the discomfort caused by pharmacological treatments.

Several similar clinical studies have shown that probiotic supplementation has no significant effect on *H. pylori* eradication rates (22, 32). However, some scholars argue that probiotics offer a potential benefit in enhancing the eradication rate of *H. pylori* infection (33, 34). In this study, the eradication rate of the two groups changed numerically, and the eradication rate of LRa05 group was higher than that of the placebo group, but the difference was not significant. Therefore, we do not recommend probiotics as a separate drug to eradicate *H. pylori*, but adding probiotics as a supplement to the original eradication therapy may be a beneficial approach.

The results showed that LRa05 can regulate the blood neutrophil count, as well as ALT and AST levels, which indicates that as a probiotic supplement, it has a certain effect on regulating inflammation, reducing neutrophil count and improving liver function. Other studies on *Lactobacillus rhamnose*-treated liver disease also support our conclusion (35, 36). Although there were significant differences in CREA levels between the two groups before and after treatment, which may be related to antibiotic treatment, CREA values were within the normal reference range for creatinine in adults (44-133 μmol/L). There were no significant changes in other blood routine and blood biochemical indexes, indicating that the safety of LRa05 as a probiotic supplement is guaranteed. To our surprise, there was a significant difference in the frequency of Pepsinogen I (PGI) abnormalities between the two groups at week 4. Pepsinogen I is strongly associated with atrophic gastritis and can be used as an indicator to assess stomach cancer risk (37, 38). Those results indicate that probiotics may have the potential to improve gastric mucosa atrophy and preventing gastric cancer.

Many previous studies have shown that probiotics play an important role in human immune regulation and regulate the expression levels of inflammatory factors such as IL-6, IL-8, TNF-α, etc (39, 40). This coincides with the conclusion of this study. The regulation of the human immune microenvironment and enhancement of immune system function are among the mechanisms that probiotics can contribute to eradicating *H. pylori* infection (10). This may explain why probiotic supplementation in this study led to improvements in gastrointestinal adverse symptoms.

Previous research studies have shown that probiotic supplementation can improve the adverse gastrointestinal symptoms caused by *H. pylori* eradication (6, 23), it can effectively relieve diarrhea, abdominal pain, nausea, constipation and other symptoms. Contrary to some other studies (13), the total GSRS score did not differ significantly between the two groups in this study after treatment. However, through further statistics, we found that after treatment, patients in the LRa05 group had significant improvements in abdominal pain, abdominal distension, acid reflux and loose stool compared with the placebo group. The positive effect of probiotics on gastrointestinal symptoms can effectively enhance patient compliance and improve the safety of eradication therapy, which is of great significance for the eradication of *H. pylori*.

Overall, after antibiotic treatment, the abundance of both beneficial and harmful bacteria decreased while in the probiotic (LRa05) group, it increased compared to the placebo group. This is consistent with some previous clinical trials (9, 25, 32). The relatively increased beneficial bacteria in the probiotic group are *Faecalibacterium*, *Lachnospira, Parabacteroides*, *Subdoligranulum*, *Phascolarctobacterium*, *Agathobacter, Fusicatenibacter*, *Alistipes*, *Coprococcus*, *Oscillibacter*, *Parasutterella*, *Megamonas*, etc. Among them, *Faecalibacterium, Parabacteroides, Phascolarctobacterium, Oscillibacter* and *Parasutterella* are all related to immune regulation and can inhibit inflammatory response (41–45). In this study, the changes of IL-6 and TNF-α levels may be related to these microbial changes. The abundance of most beneficial bacteria genera generally decreased after treatment, but the decrease of these microorganisms was smaller in patients receiving probiotics, indicating that supplementation of probiotics has a protective effect on intestinal microbiota homeostasis. Antibiotic treatment resulted in a decrease in the abundance of most bacteria, but a few species showed an increasing trend at the end of treatment. In this study, it was observed that in both the probiotic-assisted antibiotic treatment and the control (placebo) group, *Proteobacteria* showed an increasing trend at the end of treatment. *Proteobacteria* is a genus of bacteria, including some important pathogenic bacteria such as *Enterobacteriales* and *H. pylori* (46). This upward trend may indicate that antibiotic treatment has affected the microbiota balance in the gut, reducing some of the competing beneficial bacteria and thus creating conditions for the growth of potentially pathogenic bacteria such as *Proteobacteria*. This is consistent with the conclusions of some previous studies (47–49). Therefore, it is reasonable to think that antibiotic treatment can affect the diversity of gut microbiota, including beneficial and harmful bacteria. Probiotic intervention can reverse the trend of antibiotic microbiota changes, mainly by increasing the abundance of beneficial bacteria. From this perspective, it is particularly important to supplement probiotics while eradicating *H. pylori* to increase the abundance of beneficial bacteria in the gut and reduce the increase of harmful bacteria.

In summary, while LRa05 intervention demonstrated some efficacy in adjunctive *H. pylori* eradication therapy, our study faces several limitations. Firstly, our sample size was insufficient. Secondly, this study lacked uniform requirements for diagnosing *H. pylori* infection during the enrollment and screening preventing scientific statistical analysis of *H. pylori* bacterial load before and after treatment. Further analysis of ^13^C breath test values may offer deeper insights into probiotics’ eradication effects on *H. pylori*. Additionally, although we supplemented probiotics for an additional 2 weeks post-therapy, this duration might have been insufficient, resulting in insignificant changes in some indicators. Hence, extending probiotic supplementation duration could potentially yield better therapeutic outcomes.

## Conclusion

This study confirmed the effectiveness of LRa05 as a supplement for *H. pylori* eradication therapy. Although it does not improve the eradication rate, it can alleviate the adverse symptoms and intestinal microbiota disturbances caused by eradication treatment, and can also regulate the inflammatory response in the body and improve liver function.

## Data Availability

The datasets generated during this study are included in this article. The 16S rRNA gene sequence from human fecal samples is accessible in the NCBI genome database under accession PRJNA1111965.

## References

[1] Marshall BJ Warren JR. Unidentified curved bacilli in the stomach of patients with gastritis and peptic ulceration. Lancet. (1984) 1:1311–5. doi: 10.1016/s0140-6736(84)91816-6 6145023

[2] C.Wotherspoon A Ortiz-Hidalgo C Falzon M Xxxg.Isaacson P. Helicobacter pylori-associated gastritis and primary B-cell gastric lymphoma. Lancet. (1991) 338:1175–6. doi: 10.1016/0140-6736(91)92035-z 1682595

[3] Suerbaum S Michetti P. Helicobacter pylori infection. N Engl J Med. (2002) 347:1175–86. doi: 10.1056/NEJMra020542 12374879

[4] Li Y Choi H Leung K Jiang F Graham DY Leung WK. Global prevalence of Helicobacter pylori infection between 1980 and 2022: a systematic review and meta-analysis. Lancet Gastroenterol Hepatol. (2023) 8:553–64. doi: 10.1016/s2468-1253(23)00070-5 37086739

[5] Yap TW Gan HM Lee YP Leow AH Azmi AN Francois F. Helicobacter pylori eradication causes perturbation of the human gut microbiome in young adults. PloS One. (2016) 11:e0151893. doi: 10.1371/journal.pone.0151893 26991500 PMC4798770

[6] Xu W Xu L Xu C. Relationship between Helicobacter pylori infection and gastrointestinal microecology. Front Cell Infect Microbiol. (2022) 12:2022.938608. doi: 10.3389/fcimb.2022.938608 PMC9433739 36061875

[7] Liu A Wang Y Song Y Du Y. Treatment with compound Lactobacillus acidophilus followed by a tetracycline- and furazolidone-containing quadruple regimen as a rescue therapy for Helicobacter pylori infection. Saudi J Gastroenterol. (2020) 26:78–83. doi: 10.4103/sjg.SJG_589_19 32295932 PMC7279076

[8] Fallone CA Moss SF Malfertheiner P. Reconciliation of recent helicobacter pylori treatment guidelines in a time of increasing resistance to antibiotics. Gastroenterology. (2019) 157:44–53. doi: 10.1053/j.gastro.2019.04.011 30998990

[9] Ji J Yang H. Using probiotics as supplementation for helicobacter pylori antibiotic therapy. Int J Mol Sci 21. (2020) 21:1136. doi: 10.3390/ijms21031136 PMC7037652 32046317

[10] Nabavi-Rad A Sadeghi A Asadzadeh Aghdaei H Yadegar A Smith SM Zali MR. The double-edged sword of probiotic supplementation on gut microbiota structure in Helicobacter pylori management. Gut Microbes. (2022) 14:2108655. doi: 10.1080/19490976.2022.2108655 35951774 PMC9373750

[11] FitzGerald J Patel S Eckenberger J Guillemard E Veiga P Schäfer F. Improved gut microbiome recovery following drug therapy is linked to abundance and replication of probiotic strains. Gut Microbes. (2022) 14:2094664. doi: 10.1080/19490976.2022.2094664 35916669 PMC9348039

[12] Buckley M Lacey S Doolan A Goodbody E Seamans K. The effect of Lactobacillus reuteri supplementation in Helicobacter pylori infection: a placebo-controlled, single-blind study. BMC Nutr. (2018) 4:48. doi: 10.1186/s40795-018-0257-4 32153909 PMC7050722

[13] Hong Q Wang J Zhang H Liu X Liu Z. Study of the effect of Lactobacillus crispatus FSCDJY67L3 on Helicobacter Pylori eradication: a double-blind randomized controlled clinical trial. Front Immunol. (2023) 14:2023.1265995. doi: 10.3389/fimmu.2023.1265995 PMC10645133 38022520

[14] Malfertheiner P Megraud F O’Morain CA Gisbert JP Kuipers EJ Axon AT. Management of Helicobacter pylori infection-the Maastricht V/Florence Consensus Report. Gut. (2017) 66:6–30. doi: 10.1136/gutjnl-2016-312288 27707777

[15] Ding SZ Du YQ Lu H Wang WH Cheng H Chen SY. Chinese consensus report on family-based helicobacter pylori infection control and management (2021 edition). Gut. (2022) 71:238–53. doi: 10.1136/gutjnl-2021-325630 PMC8762011 34836916

[16] Hu Y Zhu Y Lu NH. Novel and effective therapeutic regimens for helicobacter pylori in an era of increasing antibiotic resistance. Front Cell Infect Microbiol. (2017) 7:2017.00168. doi: 10.3389/fcimb.2017.00168 PMC5418237 28529929

[17] Liu DS Wang YH Zeng ZR Zhang ZY Lu H Xu JM. Primary antibiotic resistance of Helicobacter pylori in Chinese patients: a multiregion prospective 7-year study. Clin Microbiol Infect. (2018) 24:780. doi: 10.1016/j.cmi.2017.11.010 29138101

[18] Sung JJY Coker OO Chu E Szeto CH Luk STY Lau HCH. Gastric microbes associated with gastric inflammation, atrophy and intestinal metaplasia 1 year after Helicobacter pylori eradication. Gut. (2020) 69:1572–80. doi: 10.1136/gutjnl-2019-319826 PMC7456733 31974133

[19] Elghannam MT Hassanien MH Ameen YA Turky EA GM EL AA EL. Helicobacter pylori and oral-gut microbiome: clinical implications. Infection. (2023) 52:289–300. doi: 10.1007/s15010-023-02115-7 PMC10954935 37917397

[20] Peng R Zhang Z Qu Y Chen W. The impact of Helicobacter pylori eradication with vonoprazan-amoxicillin dual therapy combined with probiotics on oral microbiota: a randomized double-blind placebo-controlled trial. Front Microbiol. (2023) 14:2023.1273709. doi: 10.3389/fmicb.2023.1273709 PMC10577438 37849923

[21] Ismail NI Nawawi KNM Hsin DCC Hao KW Mahmood N Chearn GLC. Probiotic containing Lactobacillus reuteri DSM 17648 as an adjunct treatment for Helicobacter pylori infection: A randomized, double-blind, placebo-controlled trial. Helicobacter. (2023) 28:e13017. doi: 10.1111/hel.13017 37614081

[22] Marinelli P Scalese G Covelli A Ruffa A Bedetti G Bruno G. Lactobacillus rhamnosus GG supplementation on eradication rate and dyspepsia in Helicobacter pylori infection treated with three-in-one bismuth quadruple therapy. Front Microbiol. (2022) 13:2022.932331. doi: 10.3389/fmicb.2022.932331 PMC9760799 36545196

[23] Plomer M Iii Perez M Greifenberg DM. Effect of Bacillus clausii Capsules in Reducing Adverse Effects Associated with Helicobacter pylori Eradication Therapy: A Randomized, Double-Blind, Controlled Trial. Infect Dis Ther. (2020) 9:867–78. doi: 10.1007/s40121-020-00333-2 PMC7680487 32897519

[24] Dong Y Han M Fei T Liu H Gai Z. Utilization of diverse oligosaccharides for growth by Bifidobacterium and Lactobacillus species and their in *vitro* co-cultivation characteristics. Int Microbiol. (2024) 27:941–52. doi: 10.1007/s10123-023-00446-x PMC11144146 37946011

[25] Gai Z Dong Y Xu F Zhang J Yang Y Wang Y. Changes in the gut microbiota composition of healthy young volunteers after administration of Lacticaseibacillus rhamnosus LRa05: A placebo-controlled study. Front Nutr. (2023) 10:2023.1105694. doi: 10.3389/fnut.2023.1105694 PMC10043436 36998912

[26] Gai Z Liao W Huang Y Dong Y Feng H Han M. Effects of Bifidobacterium BL21 and Lacticaseibacillus LRa05 on gut microbiota in type 2 diabetes mellitus mice. AMB Express. (2023) 13:97. doi: 10.1186/s13568-023-01603-1 37716924 PMC10505128

[27] Sun M Wu T Zhang G Liu R Sui W Zhang M. Lactobacillus rhamnosus LRa05 improves lipid accumulation in mice fed with a high fat diet via regulating the intestinal microbiota, reducing glucose content and promoting liver carbohydrate metabolism. Food Funct. (2020) 11:9514–25. doi: 10.1039/d0fo01720e 33063800

[28] Chen T Shao Y Zhang Y Zhao Y Han M Gai Z. *In vitro* and in *vivo* genome-based safety evaluation of Lacticaseibacillus rhamnosus LRa05. Food Chem Toxicol. (2024) 186:114600. doi: 10.1016/j.fct.2024.114600 38490350

[29] Wu T Zhang Y Li W Zhao Y Long H Muhindo EM. Lactobacillus rhamnosus LRa05 Ameliorate Hyperglycemia through a Regulating Glucagon-Mediated Signaling Pathway and Gut Microbiota in Type 2 Diabetic Mice. J Agric Food Chem. (2021) 69:8797–806. doi: 10.1021/acs.jafc.1c02925 34340304

[30] Lash RH Genta RM. Routine anti-helicobacter immunohistochemical staining is significantly superior to reflex staining protocols for the detection of helicobacter in gastric biopsy specimens. Helicobacter. (2016) 21:581–5. doi: 10.1111/hel.12315 27172813

[31] Logan RP. Urea breath tests in the management of Helicobacter pylori infection. Gut. (1998) 43 Suppl 1:S47–50. doi: 10.1136/gut.43.2008.s47 PMC1766595 9764040

[32] He C Xie Y Zhu Y Zhuang K Huo L Yu Y. Probiotics modulate gastrointestinal microbiota after Helicobacter pylori eradication: A multicenter randomized double-blind placebo-controlled trial. Front Immunol. (2022) 13:2022.1033063. doi: 10.3389/fimmu.2022.1033063 PMC9679295 36426355

[33] Liang B Yuan Y Peng XJ Liu XL Hu XK Xing DM. Current and future perspectives for Helicobacter pylori treatment and management: From antibiotics to probiotics. Front Cell Infect Microbiol. (2022) 12:2022.1042070. doi: 10.3389/fcimb.2022.1042070 PMC9732553 36506013

[34] Tang B Tang L Huang C Tian C Chen L He Z. The effect of probiotics supplementation on gut microbiota after helicobacter pylori eradication: A multicenter randomized controlled trial. Infect Dis Ther. (2021) 10:317–33. doi: 10.1007/s40121-020-00372-9 PMC7955021 33270205

[35] Vajro P Mandato C Licenziati MR Franzese A Vitale DF Lenta S. Effects of Lactobacillus rhamnosus strain GG in pediatric obesity-related liver disease. J Pediatr Gastroenterol Nutr. (2011) 52:740–3. doi: 10.1097/MPG.0b013e31821f9b85 21505361

[36] Vatsalya V Feng W Kong M Hu H Szabo G McCullough A. The beneficial effects of lactobacillus GG therapy on liver and drinking assessments in patients with moderate alcohol-associated hepatitis. Am J Gastroenterol. (2023) 118:1457–60. doi: 10.14309/ajg.0000000000002283 PMC10524173 37040544

[37] Malfertheiner P. Editorial: the non-invasive diagnosis of atrophic gastritis. Aliment Pharmacol Ther. (2017) 46:1112–3. doi: 10.1111/apt.14340 29105139

[38] Kitahara F Kobayashi K Sato T Kojima Y Araki T Fujino MA. Accuracy of screening for gastric cancer using serum pepsinogen concentrations. Gut. (1999) 44:693–7. doi: 10.1136/gut.44.5.693 PMC1727514 10205207

[39] Suez J Zmora N Segal E Elinav E. The pros, cons, and many unknowns of probiotics. Nat Med. (2019) 25:716–29. doi: 10.1038/s41591-019-0439-x 31061539

[40] Thomas CM Versalovic J. Probiotics-host communication: Modulation of signaling pathways in the intestine. Gut Microbes. (2010) 1:148–63. doi: 10.4161/gmic.1.3.11712 PMC2909492 20672012

[41] Mart416 R Rios-Covian D Huillet E Auger S Khazaal S Bermaal,ian,c.1. LG. Faecalibacterium: a bacterial genus with promising human health applications. FEMS Microbiol Rev 47. (2023) 47:fuad039. doi: 10.1093/femsre/fuad039 PMC10410495 37451743

[42] Lei Y Tang L Liu S Hu S Wu L Liu Y. Parabacteroides produces acetate to alleviate heparanase-exacerbated acute pancreatitis through reducing neutrophil infiltration. Microbiome. (2021) 9:115. doi: 10.1186/s40168-021-01065-2 34016163 PMC8138927

[43] Yang Z Su H Lv Y Tao H Jiang Y Ni Z. Inulin intervention attenuates hepatic steatosis in rats via modulating gut microbiota and maintaining intestinal barrier function. Food Res Int. (2023) 163:112309. doi: 10.1016/j.foodres.2022.112309 36596207

[44] Yang J Li Y Wen Z Liu W Meng L Huang H. Oscillospira - a candidate for the next-generation probiotics. Gut Microbes. (2021) 13:1987783. doi: 10.1080/19490976.2021.1987783 34693878 PMC8547878

[45] Chen YJ Wu H Wu SD Lu N Wang YT Liu HN. Parasutterella, in association with irritable bowel syndrome and intestinal chronic inflammation. J Gastroenterol Hepatol. (2018) 33:1844–52. doi: 10.1111/jgh.14281 29744928

[46] Taylor JA Sichel SR Salama NR. Bent bacteria: A comparison of cell shape mechanisms in proteobacteria. Annu Rev Microbiol. (2019) 73:457–80. doi: 10.1146/annurev-micro-020518-115919 31206344

[47] Becattini S Taur Y Pamer EG. Antibiotic-induced changes in the intestinal microbiota and disease. Trends Mol Med. (2016) 22:458–78. doi: 10.1016/j.molmed.2016.04.003 PMC4885777 27178527

[48] Ianiro G Tilg H Gasbarrini A. Antibiotics as deep modulators of gut microbiota: between good and evil. Gut. (2016) 65:1906–15. doi: 10.1136/gutjnl-2016-312297 27531828

[49] Korpela K Salonen A Vepsäläinen O Suomalainen M Kolmeder C Varjosalo M. Probiotic supplementation restores normal microbiota composition and function in antibiotic-treated and in caesarean-born infants. Microbiome. (2018) 6:182. doi: 10.1186/s40168-018-0567-4 30326954 PMC6192119

